# Bariatric surgery discovering unexpected silent gastric cancer: a case report

**DOI:** 10.1093/jscr/rjae209

**Published:** 2024-04-24

**Authors:** Mustafa Mohammed Taher, Mohammed A Abdalqader, Subhashini Jahanath, Nisa Nabila Nasharuddin, Yousif Nazar Yousif Al-Hamdan

**Affiliations:** Upper Gastrointestinal and Bariatric Surgery Department, Cengild Medical Centre, Bangsar South, Kuala Lumpur 59200, Malaysia; Faculty of Medicine, University of Cyberjaya, Persiaran Bestari, Cyber 11, Cyberjaya 63000, Selangor Darul Ehsan, Malaysia; Upper Gastrointestinal and Bariatric Surgery Department, Cengild Medical Centre, Bangsar South, Kuala Lumpur 59200, Malaysia; Upper Gastrointestinal and Bariatric Surgery Department, Cengild Medical Centre, Bangsar South, Kuala Lumpur 59200, Malaysia; Upper Gastrointestinal and Bariatric Surgery Department, Cengild Medical Centre, Bangsar South, Kuala Lumpur 59200, Malaysia

**Keywords:** stomach cancer, incidentally discovered, bariatric surgery

## Abstract

This research paper discusses a case in which stomach cancer was incidentally discovered during a bariatric surgery procedure. Bariatric surgery is well-known for its significant effects on weight loss and overall health enhancement, and its prevalence has been rising globally. While its primary aim is weight reduction, it also offers the chance for surgeons to detect and manage other medical conditions. In this specific case, a patient scheduled for bariatric surgery was incidentally discovered with stomach cancer, underscoring the significance of comprehensive operative assessments and vigilant monitoring during surgery.

## Introduction

Gastric cancer ranks as the fifth most common cancer globally and the fourth leading cause of cancer-related mortality as of 2020 [1]. Approximately 1.1 million cases of gastric cancer were diagnosed worldwide in 2020, with a higher incidence among males [2]. Despite its prevalence, incidental findings of gastric carcinoma before or during surgery are infrequent. For instance, a 2017 study observed incidental gastric cancers during laparoscopic bariatric surgery, estimating an incidence of 2% among 915 patients undergoing laparoscopic sleeve gastrectomy (LSG) at their institution. Importantly, all detected tumors were deemed low or very low risk for malignancy, obviating the need for adjuvant therapy post-surgery. Moreover, a 5-year follow-up revealed asymptomatic and disease-free patients, underscoring the potential for effective management better when gastric cancers are detected at an early stage [3].

Obesity, marked by an abnormal accumulation of fat, is a growing global health concern and a major contributor to illness and mortality [4]. Bariatric surgery is recognized as the most effective treatment for obesity, it is efficient in weight and Body Mass Index (BMI) reduction. In a study done in Malaysia, it was found that the average BMI 1 month pre- operation was 39.69, and 33.81 at 3 months and 27.99 at 12 months of follow-up visits [5].

Other than that, *Helicobacter pylori* (*H. pylori*) is a type of gram-negative bacillus known for its strong connection to the development of peptic ulcers and gastric cancer [6]. It is recognized as the most common chronic infection affecting people of all age groups globally, with an estimated 50% of the world’s population affected by *H. pylori* infection [7]. Notably, *H. pylori* infection is associated with gastroduodenal ulceration and is classified as a Class-I carcinogen due to its role in the formation of gastric cancer [8].

In addition to this, there is also a substantial evidence linking a higher BMI to an increased risk of cancer development. Obesity not only heightens the morbidity and mortality risks for cancer patients but also amplifies the chances of cancer progression, recurrence, and mortality. Furthermore, scientific research has identified a group of cancers termed “obesity-related cancers,” which are particularly linked to obesity [9].

This presents a case wherein stomach cancer was incidentally identified during a bariatric surgery procedure, illustrating its subsequent management. Our objective is to present an incidental gastric cancer case report and explore this management option for consideration.

## Case report

A 41-year-old female patient with a medical history of obesity and hyperlipidemia presented for a surgical consultation due to her high BMI of 39 and a decade of unsuccessful attempts to lose weight. Preoperative evaluations, including laboratory examinations and abdominal ultrasound, revealed hyperlipidemia, fatty liver, and normal other parameters.

**Figure 1:**
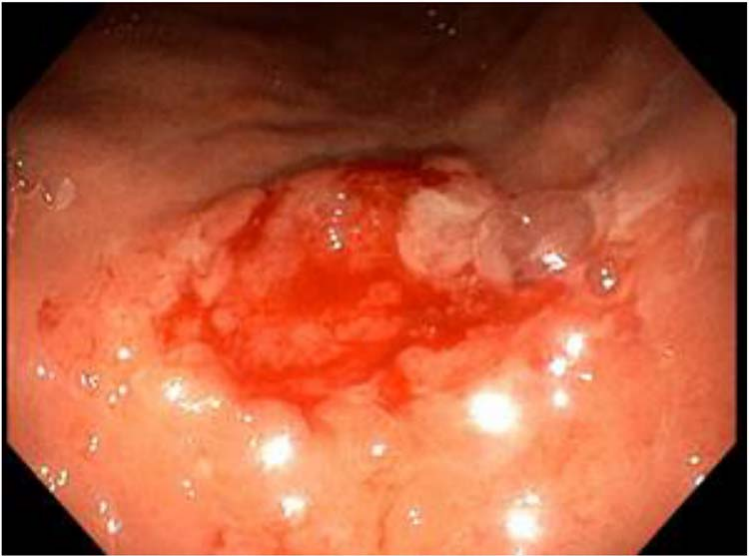
A small, raised ulcer measuring ~2 cm in diameter. Redness, irregular shape with an everted edge.

As part of our protocol, we do an endoscopic assessment on the table for better assessment and less risk of sedation in an obese patient where most of them have some sort of obstructive sleep apnea, however in patients who present with significant Gastroesophageal Reflux Disease (GERD) symptoms according to clinical assessment and using the scoring system for GERD, and if it is found to have significant reflux then we will do an endoscopic examination before surgery because this will affect the choice of type of surgery between high or low-pressure system procedure. During an upper endoscopy, a 2 cm ulcer with raised edges was seen at the pre-pyloric region within the area of the great curvature, which showed very suspicious endoscopic features of malignancy (Fig. 1). As a protocol and as being elaborated to the patient in the preoperative consultation, in these types of conditions, it is advisable to take a biopsy and defer the surgery, due to the location of the ulcer within the great curvature and the ability to remove it with the sleeve specimen with safe margin.

A discussion with the patient’s husband and agreed to proceed with the sleeve operation and trace the biopsy result and further medical action will be taken according to the biopsy results. A *H. pylori* infection was confirmed. Consequently, a diagnostic laparoscopic examination was negative and proceeded with sleeve gastrectomy (LSG) in the usual manner, the patient had a smooth recovery and was discharged home well on the next day.

**Figure 2:**
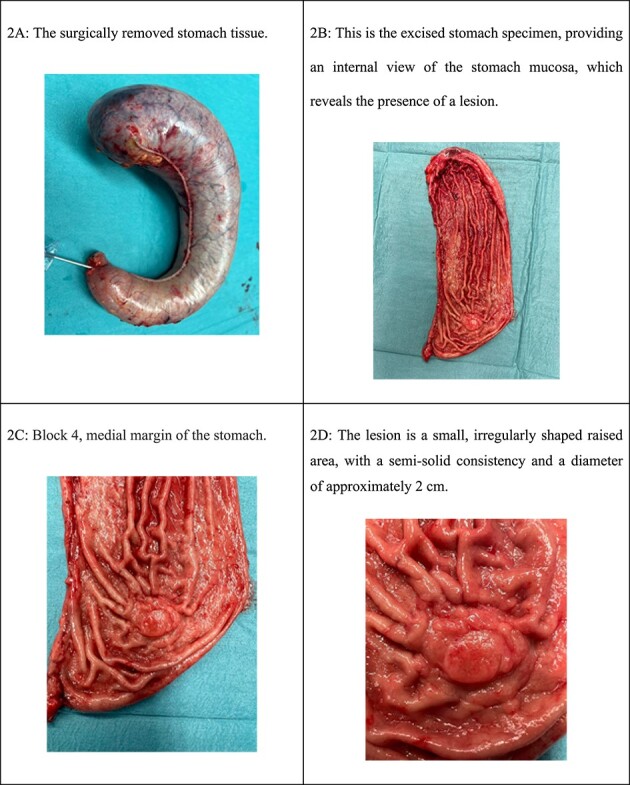
(A) The surgically removed stomach tissue. (B) This is the excised stomach specimen, providing an internal view of the stomach mucosa, which reveals the presence of a lesion. (C) Block 4, medial margin of the stomach. (D) The lesion is a small, irregularly shaped raised area, with a semi-solid consistency and a diameter of ~2 cm.

**Figure 3:**
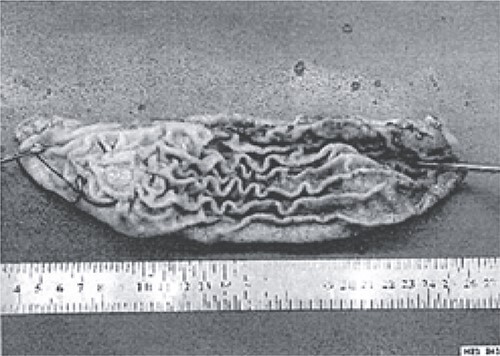
The excised stomach sample was dispatched for pathological examination.

Laparoscopic examination showed no lymphadenopathy or peritoneal disease, the removed stomach was sent to the laboratory for pathological examination and ulcers were marked with sutures as medial, lateral, proximal, and distal margins and as can be seen in Figs 2 and 3.

Histopathological examination showed *H. pylori* infection was positive. One section from the medial margin showed clusters of malignant cells with a signet ring pattern in a fibrotic stroma, along with mucin lakes containing tumor cells. This invasion mainly occurred in the submucosa, without obvious vascular or cellular invasion, and the rest of the margins were all negatives.

We do believe these types of malignant micro-infiltration will be easily missed in the normal biopsy process especially when the malignant changes are only confined to micro-clustered cells.

A staging transverse axial contrast-enhanced CT scan for thorax, abdomen, and pelvis was done and it was negative for any metastasis or lymphadenopathy.

The patient and family were informed about the findings, and a proper multidisciplinary family meeting with the radiologist and oncologist was carried out given the early disease but diffuse type signet cell, completion total gastrectomy with lymphadenectomy suggest for best prognosis and outcome (D2 total gastrectomy). The specimen from the total gastrectomy came out without any residual malignancy, and lymph nodes removed were sufficient and all negative. Patient will only need surveillance scope and monitoring in the future.

## Discussion

Historically, gastric cancer has been a leading cause of global cancer-related deaths since the 1980s. However, the landscape has evolved. Presently, it ranks 15th in the USA and Asian countries, causing ~11 140 deaths in the USA in 2019 [10]. The increased prevalence of bariatric surgeries has led to more incidental findings. A study by Chiappetta et al. from January 2010 to March 2014 identified eight patients undergoing bariatric surgery with incidental gastric cancer, equating to an incidence rate of 0.31% (3 per 1000) [11]. Importantly, identifying and addressing risk factors like *H. pylori* infection, along with dietary and environmental changes, have contributed to the decline in gastric cancer incidence.

The unexpected discovery of stomach cancer during bariatric surgery highlights the need for comprehensive preoperative assessments. This case emphasizes the limitations of routine screening methods in detecting hidden malignancies, particularly when there are no apparent clinical indications. In our patient’s case, the primary concern was morbid obesity, and she displayed no cancer-related symptoms. Typically, gastric cancer is associated with symptoms like weight loss, early satiety, and epigastric abdominal pain during the initial diagnosis which the patient presented with none [12].

Gastric cancer has two distinct types: diffuse, characterized by signet ring cell adenocarcinoma, and intestinal. Historically, the diffuse type with signet ring histology was thought to imply a worse prognosis. Recent evidence from multiple studies, however, suggests that while signet ring histology itself may not predict a poorer prognosis, it often indicates a more advanced disease stage at diagnosis. Unlike intestinal gastric cancer, which is strongly associated with long-standing gastritis caused by *H. pylori* infection, traditional risk factors are not significant contributors to diffuse-type gastric cancer development [13, 14].

Gastric cancer, especially signet ring cell carcinoma, often lacks prominent symptoms and can mimic conditions like gastroesophageal reflux disease or gastritis. In some cases, notable weight loss is the only sign, and cancer may be incidentally found via computed tomography (CT) or endoscopy [15]. Hence, a comprehensive preoperative assessment, including a detailed medical history, imaging, tissue microscopic examination, immunochemistry assay, and possibly endoscopy, is crucial to avoid overlooking important issues.

In managing these tumors, surgical resection with comprehensive lymphadenectomy is essential. This approach aids in assessing prognosis, preventing stage migration, and guiding therapeutic decisions [15].

Intraoperative vigilance is vital in bariatric surgery to detect unexpected findings, like suspected malignancies, which can prompt changes in the surgical plan, extended resection, or collaboration with a multidisciplinary team for optimal patient care. Early diagnosis and intervention, akin to a pivotal climax, have the potential to significantly enhance outcomes for patients with incidental stomach cancer.

## Conclusion

In summary, this compelling case report underscores the significance of thorough preoperative evaluations and surgical vigilance in uncovering unexpected findings like stomach cancer during bariatric surgery. It highlights the limitations of conventional screening methods and advocates for a multidimensional approach to patient care. It also elucidates the intricate connection between *H. pylori* infection and gastric cancer, emphasizing the critical need for comprehensive research and preventive strategies in this context.

As this impactful case report concludes, it leaves a lasting impression, urging healthcare practitioners to embrace the intriguing possibilities presented by incidental findings in the context of bariatric surgery.

## References

[1] Sung H , Ferlay J, Siegel RL, et al. Global cancer statistics 2020: GLOBOCAN estimates of incidence and mortality worldwide for 36 cancers in 185 countries. CA Cancer J Clin 2021;71:209–49. 10.3322/caac.21660. 33538338

[2] Morgan E , Arnold M, Camargo MC, et al. The current and future incidence and mortality of gastric cancer in 185 countries, 2020-40: a population-based modelling study. EClinicalMedicine 2022;47:101404. 10.1016/j.eclinm.2022.101404. 35497064 PMC9046108

[3] Viscido G , Signorini F, Navarro L, et al. Incidental finding of gastrointestinal stromal tumors during laparoscopic sleeve gastrectomy in obese patients. Obes Surg 2017;27:2022–5. 10.1007/s11695-017-2583-z. 28185152

[4] Öner Rİ , Özdaş S. Histopathological findings in morbid obese patients undergoing laparoscopic sleeve gastrectomy: does *H. pylori* infection effective on pathological changes. Obes Surg 2018;28:3136–41. 10.1007/s11695-018-3250-8. 29663251

[5] Taher MM , Abdalqader MA, Jahanath S, et al. Bariatric surgeries: outcome throughout an annum at a specialist center in Malaysia. PloS One 2023;18:e0285196. 10.1371/journal.pone.0285196. 37159461 PMC10168565

[6] Emile SH , Elshobaky A, Elbanna HG, et al. Helicobacter pylori, sleeve gastrectomy, and gastroesophageal reflux disease; Is there a relation. Obes Surg 2020;30:3037–45. 10.1007/s11695-020-04648-4. 32358686

[7] Baillargeon D , Greenblatt M, Côté M, et al. Prevalence of helicobacter pylori infection in bariatric surgery patients. Obes Surg 2023;33:2132–8. 10.1007/s11695-023-06638-8. 37202576

[8] Shahraki MS , Pouramini A, Heydari Y, Shahabi Shahmiri S. Does bariatric surgery change the recurrence of helicobacter pylori infection. Obes Surg 2021;31:4210–2. 10.1007/s11695-021-05507-6. 34089440

[9] Valadares EC , Gestic MA, Utrini MP, et al. Pre-operative screening of helicobacter pylori in bariatric patients: is histopathological analysis necessary? Arq Gastroenterology 2022;59:275–80. 10.1590/S0004-2803.202202000-49. 35830041

[10] Cancer stat facts. Stomach cancer [Nov;2019]; http://seer.cancer.gov/statfacts/html/stomach.html 2018

[11] Chiappetta S , Theodoridou S, Stier C, Weiner RA. Incidental finding of GIST during obesity surgery. Obes Surg 2015;25:579–83. 10.1007/s11695-015-1571-4. 25596937

[12] Wanebo HJ , Kennedy BJ, Chmiel J, et al. Cancer of the stomach. A patient care study by the American College of Surgeons. Ann Surg 1993;218:583–92. 10.1097/00000658-199321850-00002. 8239772 PMC1243028

[13] Bamboat ZM , Tang LH, Vinuela E, et al. Stage-stratified prognosis of signet ring cell histology in patients undergoing curative resection for gastric adenocarcinoma. Ann Surg Oncol 2014;21:1678–85. 10.1245/s10434-013-3466-8. 24394986

[14] Kim DY , Park YK, Joo JK, et al. Clinic pathological characteristics of signet ring cell carcinoma of the stomach. J Surg 2004;74:1060–4. 10.1111/j.1445-1433.2004.03268.x 15574148

[15] Pernot S , Voron T, Perkins G, et al. Signet-ring cell carcinoma of the stomach: impact on prognosis and specific therapeutic challenge. World J Gastroenterology 2015;21:11428–38. https://doi.org/10.3748/wjg. V21.i40.11428. 10.3748/wjg.v21.i40.11428 PMC4616218 26523107

